# Low back pain trends attributable to high body mass index over the period 1990–2021 and projections up to 2036

**DOI:** 10.3389/fnut.2024.1521567

**Published:** 2025-01-21

**Authors:** Jiling Zhang, Baodong Wang, Congying Zou, Tianyi Wang, Lihui Yang, Yu Zhou

**Affiliations:** ^1^Department of Clinical Laboratory, Beijing Shunyi District Hospital, Beijing, China; ^2^Department of Orthopedics, Beijing Chaoyang Hospital, Capital Medical University, Beijing, China

**Keywords:** low back pain, body mass index, global burden of disease, disability-adjusted life-years, socio-demographic index

## Abstract

**Background:**

High body mass index (BMI) is a crucial determinant in low back pain (LBP) incidence and progression. However, the effect of increased BMI on LBP has been largely overlooked at the global, regional, and national levels. This research aimed to use data from the 2021 global burden of disease (GBD) study to determine trends associated with LBP due to high BMI from 1990 to 2021, thereby providing evidence for developing targeted policies.

**Methods:**

Epidemiological data on the association between high BMI and LBP is obtained from the GBD 2021. Disability-adjusted life-years (DALYs) attributable to high BMI-related LBP are stratified by year, age, country, and socio-demographic index (SDI). The estimated annual percentage change (EAPC) was calculated to evaluate the trends from 1990 to 2021. A Bayesian age-period cohort (BAPC) model was used to assess the corresponding trends from 2022 to 2036. Additionally, statistical models, such as decomposition analysis and frontier analysis, were used.

**Results:**

According to the GBD 2021, the number of DALYs caused by LBP attributed to high BMI reached 8,363,759 in 2021, which is an increase of 170.97% since 1990. The age-standardized rate of disability-adjusted life years (ASDR) for LBP caused by high BMI has been increasing from 1990 to 2021, with an EAPC of 1.14%. Among the five SDI regions, ASDR has increased. High-income North Americans exhibited the highest risk of LBP caused by high BMI, with Hungary being the most affected. Frontier analysis highlights the urgent need for intervention in countries such as the Netherlands, Germany, and Canada. Finally, the burden of LBP related to high BMI will continue to rise from 2022 to 2036.

**Conclusion:**

Between 1990 and 2021, there was a global increase in lower back pain due to high BMI, with a projected continuation of this trend. Monitoring BMI is crucial for developing region-specific and national strategies, and research emphasizes the urgency of reducing the health burden of high BMI and improving the quality of life for the global population.

## 1 Introduction

Low back pain (LBP), a prevalent musculoskeletal condition, has appeared as a significant global public health challenge (1). Definition Clinically, LBP is defined as pain in the lower back region, from the lower edge of the twelfth rib to the gluteal fold, with or without pain radiating to one or both lower limbs, lasting for at least one day (2). The International Classification of Diseases, Ninth Revision, code for this condition is 724, specifically indicating LBP. The global burden of disease (GBD) study determines LBP as a major contributor to disability globally (3). In the global context of disability-adjusted life years (DALYs), LBP is the ninth leading cause, yet it tops the list in terms of years lived with disability (YLDs), representing 2.5% and 7.41% of the total global DALYs and YLDs, respectively (4). The global incidence of back pain across all age groups in 2019 was documented at 2,887.97 cases per 100,000 people, indicating a 3.49% increase since 1990 (5). This upward trajectory emphasizes the substantial effect of LBP on individual health and its considerable burden on social welfare and healthcare systems. The consequences of LBP are far-reaching; it is intricately related to chronic disability, declined quality of life, and a notable healthcare expenditure escalation. This includes the extensive use of medical resources, including pharmaceuticals, medical consultations, radiological examinations, and physical therapy (6–9). Consequently, the economic and social burden of LBP is increasing, posing a formidable challenge to individuals, society, and healthcare systems alike. Addressing this issue warrants a concerted effort to mitigate the effect of LBP and to develop sustainable strategies that alleviate the strain on both individuals and healthcare resources.

The GBD study is a worldwide health research initiative aimed at assessing the impact of various diseases, injuries, and risk factors on human health globally, providing timely, effective, and relevant health outcome assessments (10, 11). The GBD data collection process quantifies the severity of all principal diseases, risk factors, and intermediate clinical outcomes, facilitating comparisons over various timeframes, among diverse populations, and across a range of health concerns (12). In addition, it supports the creation of effective health policies, the forecasting of future health trends, and the timely surveillance of global health status, supplying a wealth of resources for global health research and policy development (13–15).

According to the definitions provided by the GBD study, for adults aged 20 and above, a high body mass index (BMI) is defined as exceeding the range of 20 to 25 kg/m^2^ (16). In the case of children and teenagers aged 1 to 19, their BMI is classified according to the criteria established by the International Obesity Task Force (IOTF): a BMI between 18.02 and 25 kg/m^2^ is considered overweight, and a BMI above the 19.81 to 30 kg/m^2^ range is classified as obese (17). Increased BMI levels are associated with an increased risk of numerous health conditions, including cardiovascular diseases, metabolic syndrome, chronic kidney disease, abnormal blood lipid levels, hypertension, specific cancer types, obstructive sleep apnea, osteoarthritis, and depression (18–21). These diseases present significant health difficulties for patients and impose a substantial economic strain on healthcare systems. The medical costs for individuals with high BMI are approximately 30% higher than for those with a normal BMI (22). A more than two-and-a-half-fold increase in global deaths and DALYs due to high BMI has been reported from 1990 to 2021. During this period, the age-standardized DALYs rate for women increased by 21.7%, whereas it exhibited a more pronounced rise for men, amounting to 31.2% (23).

Previous research has indicated BMI and excessive physical activity as key contributors to persistent postpartum LBP (24). Additionally, studies have revealed a significant correlation between BMI and the risk of all types of back pain, with an especially close association with LBP (25). Studies by Japanese researchers have further established a significant positive correlation between BMI and LBP persistence (26). High BMI is a modifiable risk factor adjusted through changes in behavior, but its significance remains unrecognized in the study of musculoskeletal diseases, especially in research related to LBP. We need to obtain precise and timely quantitative data at both regional and global levels to effectively tackle this challenge. Hence, we used data from the 2021 GBD to conduct a thorough analysis of the trends in DALYs from 1990 to 2021, both globally and for each country, and we also forecasted future disease burdens. These research finding not only add to the existing research but also significantly guide the formulation and promotion of preventive strategies for LBP induced by high BMI. We hope that these research efforts will help reduce the effect of high BMI on the burden of lower back pain and thus increase the global public health standards.

## 2 Materials and methods

### 2.1 Data source

We obtained data on LBP attributed to high BMI from the GBD 2021 results tool on the GHDx platform (ghdx.healthdata.org/gbd-results-tool). To date, the GBD 2021 has gathered data on 371 diseases and injuries, including incidence, prevalence, mortality, YLDs, and DALYs, across 204 countries and territories from 1990 to 2021, as well as data on 88 risk factors (24). The nuanced methodologies underpinning the GBD 2021 and its comparative risk assessment for increased BMI have been elaborated in other sources, whereas our retrieved dataset encompasses the following (27): (1) global, regional, and nation-specific age-standardized DALYs rates for LBP related to high BMI across three gender groups from 1990 to 2021; (2) corresponding global, regional, and national age-standardized DALYs rates for high BMI-attributed LBP among three gender groups from 1990 to 2019; (3) socio-demographic index (SDI) scores for 204 countries and territories ranging from 1990 to 2019; and (4) anticipated shifts in global population demographics from 2020 to 2050. The GBD database, which is an open-source epidemiological database, aims to quantify global health and disease trends. Ethical approval from an institutional review board was waived for this study because of the public nature of the GBD 2021 data (28).

### 2.2 Definition

The burden of LBP attributable to high BMI is evaluated based on the level of national development, as indicated by the SDI (29). The SDI is a composite indicator that combines three separate indicators: the total fertility rate for women under 25 years of age, the average educational attainment for individuals aged ≥ 15 years, and the per capita income with a lag distribution. The 204 countries and regions are categorized based on these criteria into five groups according to their SDI values: low SDI (< 0.45), lower-middle SDI (0.45 to < 0.61), middle SDI (0.61 to < 0.69), upper-middle SDI (0.69 to < 0.80), and high SDI (≥ 0.80).

### 2.3 Statistical analysis

In this study, we tracked trends by the estimated annual percentage change (EAPC) in age-standardized DALYs (ASDR) from 1990 to 2021. The EAPC was estimated according to a linear model expressed as y = α+βx+ε, where y denotes the natural logarithm of ASDR, x indicates the corresponding year, and β represents the linear regression coefficient. The EAPC is determined based on this model using the formula EAPC = 100 × (exp(β)−1). An EAPC and its 95% confidence interval (CI) of > 0 indicate an increasing ASDR, < 0 denotes a decreasing ASDR, and inclusion of 0 within the 95% CI represents no significant variation in the rate (30). We conducted a decomposition analysis to determine the individual contributions of various independent factors affecting DALYs attributed to high BMI-related LBP, including population age structure, population growth, and epidemiological shifts. We assessed the extent to which specific factors affect epidemiological trends by comparing ideal scenario outcomes with real-world outcomes while controlling other variables (31, 32). Additionally, the correlation between the SDI and age-standardized DALYs was calculated using Pearson’s correlation coefficient. Frontier analysis was conducted to evaluate the ideal levels of DALYs at the respective SDI levels for 204 countries and territorieand to pinpoint the regions with the starkest disparities (33, 34). Therefore, we used the Integrated Nested Laplace Approximation (INLA) framework and Bayesian Age-Period-Cohort (BAPC) models to project future trends (35). The INLA framework, when combined with the BAPC model, is used to approximate marginal posterior distributions, effectively avoiding the mixing and convergence issues associated with traditional Bayesian methods that rely on Markov Chain Monte Carlo sampling techniques (36). All BAPC analyses were conducted using the R packages “BAPC” (version 0.0.36) and “INLA.” R version 4.4.0 was used for statistical analyses and data visualization, considering a *P*-value of < 0.05 as statistically significant.

## 3 Results

### 3.1 Global trends in DALYs due to LBP attributed to high BMI

**FIGURE 1:**
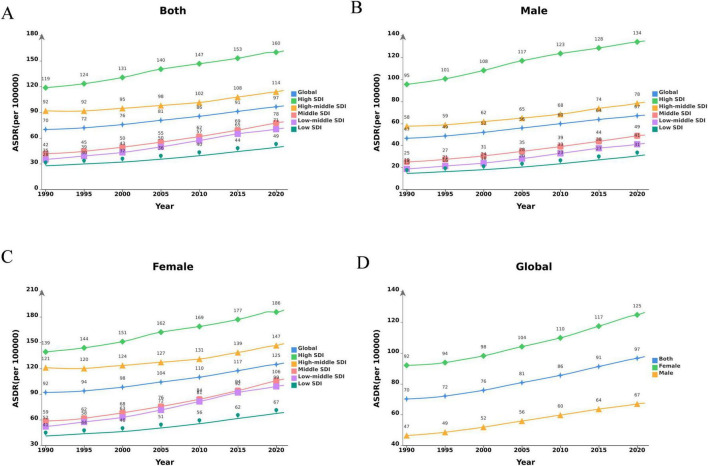
Changes in the ASDR of LBP attributable to high BMI globally and in different SDI regions from 1990 to 2021. **(A)** Both genders; **(B)** male; **(C)** female; **(D)** global. DALYs, disability-adjusted life-years; LBP, low back pain; BMI, body mass index; SDI, socio demographic index.

**FIGURE 2:**
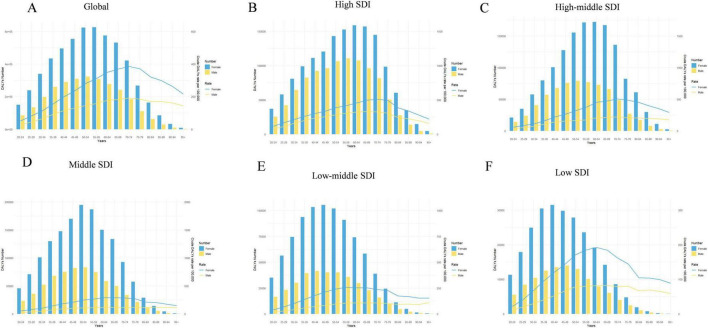
Age-standardized numbers and rates of DALYs of LBP attributable to high BMI by age and gender, in 2021. **(A)** Global; **(B)** high SDI; **(C)** high–middle SDI; **(D)** middle SDI; **(E)** low–middle SDI; **(F)** low SDI. DALYs, disability-adjusted life-years; LBP, low back pain; BMI, body mass index; SDI, socio demographic index.

The number of global DALYs attributed to LBP due to high BMI more than tripled from 1990 to 2021, increasing from 3,086,573 in 1990 to 8,363,759 in 2021. Concurrently, the ASDR increased from 70.22 to 97.66 per 100,000 people, with a global EAPC value of 1.14 (95% CI: 1.11–1.17) (Table 1 and Figure 1). The increasing trend in ASDR and DALYs was slightly higher for males than for females, although the burden among females was significantly greater than among males. The highest ASDR in 2021 was observed in high SDI regions, peaking at 161.80 (95% CI: 16.00–332.59), with a decrease in ASDR as SDI levels. The disease burden was greater in high SDI regions, but a more significant increasing trend in ASDR was observed in regions with medium SDI (EAPC: 2.18 [95% CI: 2.13–2.22]), medium–low SDI (EAPC: 2.46 [95% CI: 2.41–2.52]), and low SDI (EAPC: 1.98 [95% CI: 1.92–2.04]). Figure 2 illustrates the distributional differences in DALYs among various age groups in 2021 across the globe and in countries with different SDI levels. A skewed distribution of DALYs across age groups was evident in medium–low SDI and low SDI regions, whereas other regions demonstrated an approximately normal distribution. The highest DALYs rates in high SDI regions were observed in women aged 70–74 years and men aged 65–69 years. The peak DALYs rates in the 55–59-year age group for women and in the 60–64-year age group for men in medium–low SDI regions. Additionally, the crude DALYs rates for all age groups significantly increased in all SDI regions between 1990 and 2021 (Supplementary Figure 1).

**TABLE 1:** DALYs and ASDR of low back pain attributable to high body mass index in 1990 and 2021, with EAPC from 1990 to 2021.

Locations	Gender	1990		2021		1990–2021
		NO. DALYs cases (95% CI)	ASDR per 100,000 (95% CI)	NO. DALYs cases (95% CI)	ASDR per 100,000 (95% CI)	EAPC NO. (95% CI)
Global	Both	3,086,573.08 (312,559.12–6,484,427.06)	70.22 (7.14–146.48)	8,363,759.33 (840,306.54–17,424,821.68)	97.66 (9.78–204.00)	1.14 (1.11–1.17)
	Female	2,072,340.09 (207,876.58–4,368,722.69)	92.01 (9.25–193.20)	5,541,374.25 (556,565.96–11,636,895.13)	126.29 (12.63–266.01)	1.09 (1.05–1.14)
	Male	1,014,232.99 (104,682.54–2,115,704.37)	46.57 (4.84–96.25)	2,822,385.08 (283,740.58–5,786,405.47)	67.56 (6.78–138.87)	1.29 (1.27–1.31)
High SDI	Both	1,218,421.82 (119,569.00–2,549,631.15)	118.84 (11.60–248.80)	2,478,626.07 (249,211.24–5,091,840.30)	161.80 (16.00–332.59)	1.06 (1.02–1.09)
	Female	754,546.81 (73,813.37–1,593,850.86)	139.3 (13.51–294.20)	1,475,151.06 (147,999.07–3,016,875.75)	188.09 (18.71–384.24)	1.02 (1–1.05)
	Male	463,875.01 (45,755.64–975,889.32)	95.48 (9.41–201.07)	1,003,475.01 (101,212.17–2,050,414.58)	134.88 (13.40–277.88)	1.19 (1.13–1.24)
High-middle SDI	Both	956,976.62 (96,319.02–2,014,411.50)	91.90 (9.24–192.00)	2,086,188.17 (212,179.44–4,263,358.45)	115.59 (11.61–238.00)	0.78 (0.72–0.84)
	Female	672,248.56 (67,097.13–1,408,160.29)	120.96 (12.04–252.65)	1,409,149.57 (143,906.54–2,869,424.09)	148.93 (15.00–306.13)	0.70 (0.64–0.77)
	Male	284,728.06 (29,221.89–601,696.97)	57.57 (5.93–119.89)	677,038.60 (68,272.90–1,403,388.71)	79.20 (7.91–164.69)	1.09 (1.04–1.13)
Middle SDI	Both	547,306.42 (57,862.32–1,153,131.65)	41.92 (4.49–87.56)	2,205,381.99 (219,659.90–4,678,252.07)	79.10 (7.86–168.24)	2.18 (2.13–2.22)
	Female	380,056.52 (39,470.20–803,844.29)	58.73 (6.17–123.38)	1,528,876.93 (152,268.32–3,244,438.81)	107.66 (10.69–228.98)	2.08 (2.02–2.13)
	Male	167,249.89 (18,392.11–349,855.98)	25.12 (2.81–51.88)	676,505.05 (67,391.58–1,425,828.90)	49.50 (4.93–104.76)	2.32 (2.29–2.34)
Low-middle SDI	Both	276,060.91 (29,215.80–573,856.68)	35.21 (3.78–72.71)	1,227,064.64 (122,732.05–2,554,600.60)	71.69 (7.24–148.95)	2.46 (2.41–2.52)
	Female	201,500.19 (20,758.21–420,454.86)	52.22 (5.45–107.58)	874,755.01 (87,518.11–1,814,784.82)	100.42 (10.13–207.43)	2.28 (2.22–2.33)
	Male	74,560.72 (8,457.60–153,202.66)	18.78 (2.16–38.06)	352,309.62 (35,130.01–745,814.35)	41.96 (4.25–88.69)	2.76 (2.7–2.82)
Low SDI	Both	82,343.43 (9,051.79–165,429.39)	27.81 (3.09–55.76)	356,409.63 (35,002.73–729,956.95)	50.30 (5.07–103.18)	1.98 (1.92–2.04)
	Female	60,496.37 (6,399.94–123,596.99)	41.21 (4.41–84.08)	246,927.64 (24,106.67–508,524.41)	68.60 (6.85–141.10)	1.71 (1.65–1.77)
	Male	21,847.05 (2,593.80–43,735.72)	14.72 (1.76–29.09)	109,481.99 (10,883.56–221,900.29)	31.49 (3.21–63.91)	2.53 (2.48–2.58)

No., number; CI, confidence interval; EAPC, estimated annual percentage change; DALYs, disability-adjusted life-years; ASDR, age-standardized DALYs rate; SDI, socio demographic index.

### 3.2 Global assessment of the disease burden of LBP caused by high BMI in 2021

**FIGURE 3:**
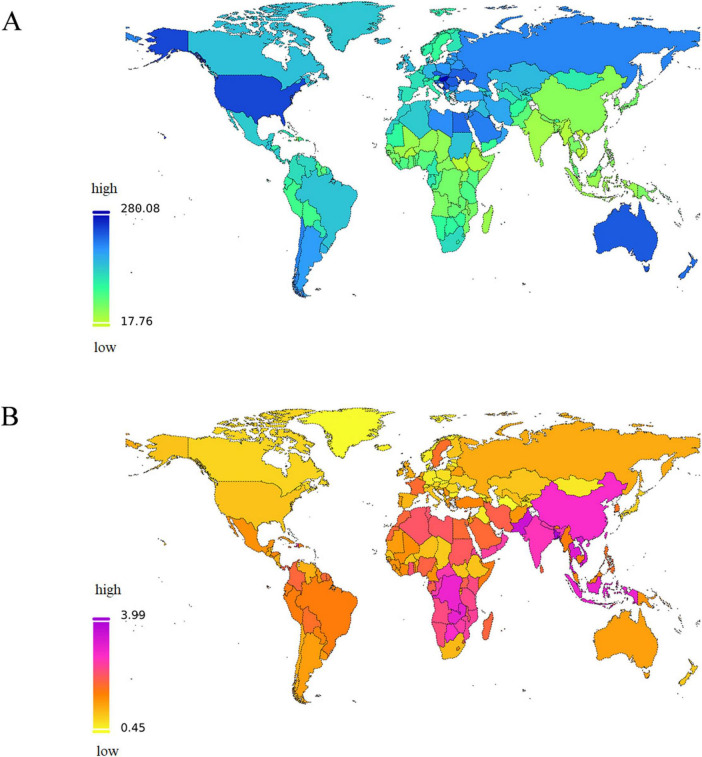
The global disease burden of LBP attributable to high BMI for both sexes combined in 204 countries and territories. **(A)** The spatial distribution of LBP ASDR attributable to high BMI in 2021. **(B)** The EAPC in LBP ASDR attributable to high BMI from 1990 to 2021. DALYs, disability-adjusted life-years; ASDR, age-standardized DALYs rate; EAPC, estimated annual percentage; LBP, low back pain; BMI, body mass index; SDI, socio demographic index.

The crude DALYs rates demonstrated an increasing trend not only from young individuals to the elderly but also from high SDI regions to low SDI regions in 2021 (Supplementary Figure 2). A world map illustrates the high heterogeneity of ASDR attributed to high BMI-related LBP and the corresponding EAPC across various countries and regions in 2021 (Figure 3). Notably high ASDR were determined in several countries in the South Pacific, as well as in the majority of countries in North America and Northern Europe (Figure 3A). The disease burden in nearly every country underwent an increase in terms of EAPC values to some extent from 1990 to 2021, with the most notable spikes in Cambodia and North Korea (Figure 3B and Supplementary Table 1).

### 3.3 Correlation analysis

**FIGURE 4:**
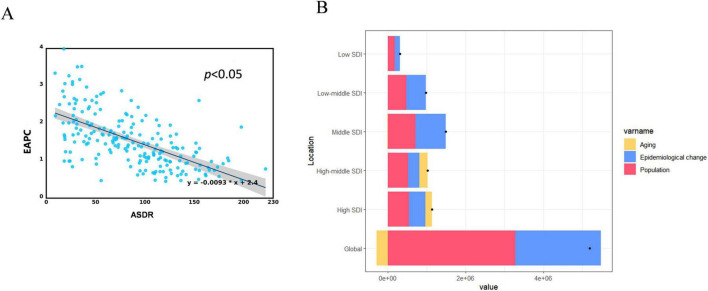
Correlation analysis and decomposition analysis. **(A)** Correlation between EAPC and ASDR of LBP attributed to high BMI in 1990. **(B)** Changes DALYs according to population-level determinants of aging, population growth, and epidemiological change from 1990 to 2021 at the global level and by SDI quintile. DALYs, disability-adjusted life-years; ASDR, age-standardized DALYs rate; EAPC, estimated annual percentage; LBP, low back pain; BMI, body mass index; SDI, socio demographic index.

The baseline prevalence of LBP attributed to high BMI was indicated by the ASDR in 1990. Our study determined a significant negative correlation between the EAPC and ASDR (Figure 4A; Pearson’s *r* = −0.61; *P* < 0.001).

### 3.4 Decomposition analysis

This study conducted a decomposition analysis of the original DALYs using data from 1990 to 2021 to evaluate the effect of aging, population growth, and epidemiological changes on the burden of LBP attributed to high BMI. Population growth and changes in epidemiology on a global scale were responsible for 63.08% and 42.51% of the increased burden of LBP attributed to high BMI, respectively. The effect of changes in epidemiology was most pronounced in regions with middle SDI, constituting 53.13%. The contributions in high, low-middle, high-middle, and low SDI regions were 37.12%, 51.05%, 28.26%, and 41.88%, respectively. The contribution of demographic aging was predominantly noticeable in higher SDI regions (high-middle SDI [20.35%], high SDI [14.8%], middle SDI [−0.47%], low-middle SDI [−0.08%], and low SDI [−0.65%]). The influence of population growth was uniformly comparable across the SDI regions (Figure 4B and Supplementary Table 2).

### 3.5 Trends of LBP attributed to high BMI in SDI regions or countries

**FIGURE 5:**
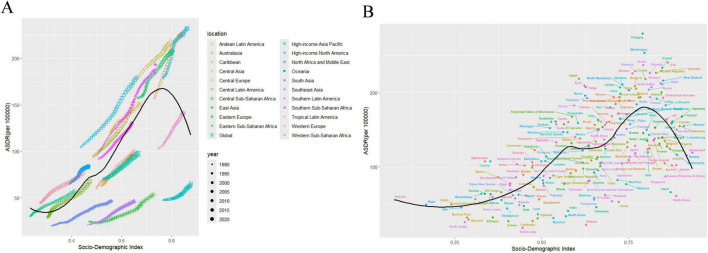
Association between age-standardized LBP attributed to high BMI DALYs and SDI. **(A)** Trend in ASDR of LBP attributed to high BMI among/across 21 regions and global based on SDI in 2021. **(B)** Trend in ASDR of LBP attributed to high BMI among/across 204 countries based on SDI in 2021. ASDR, age-standardized DALYs rate; LBP, low back pain; BMI, body mass index; SDI, socio demographic index.

The overall disease burden is increasing as the economy improves. The burden of LBP attributed to high BMI continues to increase when the SDI value is < 0.75 in 21 regions. The burden peaks when the SDI reaches 0.75. The burden begins to decline after exceeding 0.75. Regions, such as high-income North America, Australasia, and Central Europe, have a burden that is higher than anticipated, whereas East Asia, Southeast Asia, and high-income Asia-Pacific regions exhibit a burden that is lower than expected (Figure 5A). The burden of LBP caused by high BMI exceeds expectations in Hungary, Montenegro, Serbia, and Egypt, whereas the burden is below expectations in Timor-Leste, Vietnam, China, and South Korea (Figure 5B).

### 3.6 Frontier analysis

**FIGURE 6:**
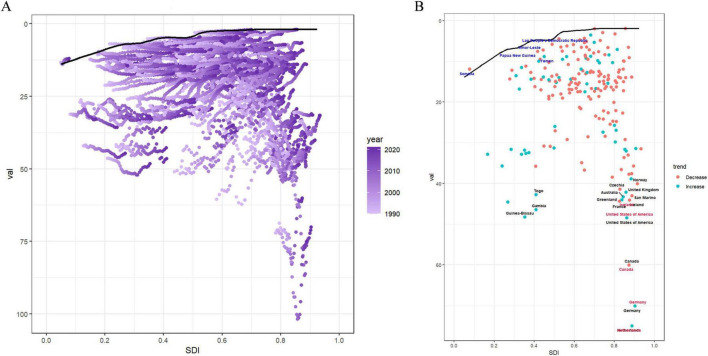
Frontier analysis results. **(A)** Illustrates leading-edge analysis based on ASDR and SDI from 1990 to 2021. Color levels range from light purple (1990) to dark purple (2021). The solid black line depicts the boundary. **(B)** Illustrates the leading-edge analysis based on ASDR and SDI in 2021. Solid black lines represent borders and dots represent countries and regions. The top 15 countries and territories with the largest effective differences are marked in black. Examples of border countries with low SDI (< 0.5) and low effective variance are marked in blue, and examples of countries and regions with high SDI (> 0.85) and relatively high effective variance are marked in red. Red dots indicate a decline in ASDR, while blue dots indicate an increase between 1990 and 2021. ASDR, age-standardized DALYs rate; SDI, socio demographic index.

This study used data from 1990 to 2021 and conducted a frontier analysis according to ASDR and SDI, considering the national development level, to investigate the potential for improvement in the burden of LBP attributed to high BMI (Figure 6A and Supplementary Table 7). The analysis identified 15 countries with the most significant actual potential for improvement. Nations, including the Netherlands, Germany, Canada, the United States of America, Guinea-Bissau, Greenland, Iceland, France, Gambia, Australia, San Marino, the United Kingdom, Czechia, Norway, and Togo, demonstrated higher ASDR compared to other countries with similar socio-demographic conditions. Border countries with a low SDI (< 0.5), such as Somalia, the Lao People’s Democratic Republic, Timor-Leste, Papua New Guinea, and Yemen, signal an urgent requirement for targeted health interventions. Conversely, countries, such as the Netherlands, Germany, Canada, the United States of America, and Iceland, despite their high SDI (> 0.85), exhibited a relatively high effective difference in development levels, indicating room for improvement in managing the burden of LBP due to high BMI (Figure 6B and Supplementary Tables 3–6).

### 3.7 Global disease burden of LBP attributed to high BMI forecast

**FIGURE 7:**
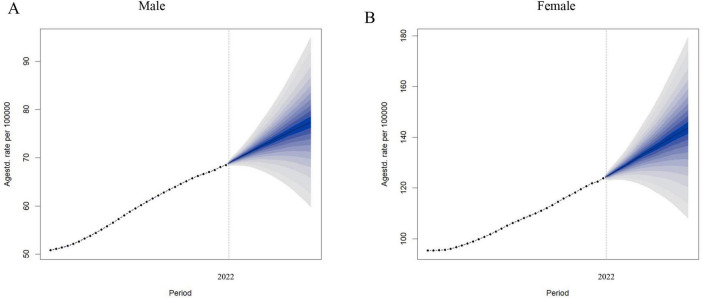
Observed and predicted trends of ASDR of LBP attributed to high BMI by sex globally from 1990 to 2036 using the BAPC model. **(A)** ASDR of male. **(B)** ASDR of female. ASDR, age-standardized DALYs rate; LBP, low back pain; BMI, body mass index; BAPC, Bayesian age-period-cohort.

We employed the BAPC model to project a significant increase in the ASDR for LBP attributed to high BMI among both males and females from 2021 to 2036 using the comprehensive GBD data spanning from 1990 to 2021. More specifically, globally, the ASDR for LBP due to high BMI in men is projected to reach 77.38 per 100,000 people in 2036 (Figure 7A). Similarly, ASDR for women is projected to climb to 143.77 per 100,000 people by 2036 (Figure 7B and Supplementary Table 7).

## 4 Discussion

Numerous studies confirmed obesity as a significant risk factor for LBP (37, 38). Currently, we use the BMI as the standard for measuring obesity (39). Individuals with obesity frequently have higher adipokine levels in their bodies, such as leptin in the serum. This hormone affects cellular metabolism, increasing proteases and nitric oxide (NO) production. Furthermore, introducing leptin into an inflammatory environment exacerbates its harmful effects, promoting inflammatory cytokine production, which may be one of the mechanisms causing back pain (40). Research indicates that patients with LBP with elevated BMI, especially women, exhibit more substantial fat infiltration in the paraspinal muscles at the thoracolumbar junction and more severe pathologies in the lower lumbar region (41). Hashem et al. (42) indicated that systemic inflammation may bridge the gap between a lack of physical activity and obesity in the etiology of chronic non-specific LBP. Moreover, a correlation between increasing BMI and increased disability was observed in LBP sufferers. Patients with severe obesity (II–III) do not demonstrate marked improvements, whereas some improvement in the disability is noted in overweight patients post-treatment (43). These results emphasize the strong association between high BMI or obesity and LBP pathogenesis.

Studies have revealed that various individual factors, including poor health status and physical and psychological stress, significantly increase the risk of LBP (44). The GBD 2019 estimates indicated that the DALYs for LBP are affected by three major risk factors: smoking accounts (15.7%), high BMI (6.7%), and occupational ergonomic factors (24%). Notably, the impact of high BMI is particularly significant in high-income North American regions, representing 11.4%. A higher proportion of LBP DALYs is attributed to high BMI in females when examining gender differences (45). The global mortality and DALYs attributed to high BMI have more than doubled from 1990 to 2017. The age-standardized DALYs rate associated with high BMI increased by a mere 12.7% in females, but it surged by 26.8% in males. Lower back pain ranked sixth among the primary causes contributing to high BMI-related DALYs in 2017. The age-standardized rate of DALYs demonstrated an increasing pattern in areas with the lowest SDI from 1990 to 2017 (46). A substantial increase in the global age-standardized DALYS rate due to high BMI, with an annual increment of 1.8% in exposure to high BMI, by 2021 (14). Consequently, the cumulative risk of LBP is on an upward trajectory alongside the growing burden of high BMI.

Previous research has already emphasized that both men and women have demonstrated a significant increase in deaths and DALYs due to high BMI from 1990 to 2021 (24). Our results align with this, indicating an increase in the ASDR of LBP caused by high BMI for both genders over 21 years, with similar increments observed. However, the ASDR for LBP due to high BMI in women (126.29 per 100,000) notably exceeded that in men by 2021 (67.56 per 100,000). These gender differences highlight the importance of formulating public health strategies that are tailored to each gender to tackle the expanding global implications of high BMI. The reasons for such gender discrepancies may include differences in pelvic architecture between women and men, as well as variations in hormonal levels during women’s menstrual cycles, pregnancy, and menopause, which could affect the functionality of ligaments and muscles, thereby increasing the risk of back pain (47). Moreover, the processes of pregnancy and childbirth may cause lower back muscle and ligament injuries (48). Women may be more susceptible to psychological factors, such as anxiety and depression, which could aggravate back pain perception (49). Finally, women’s potentially higher pain sensitivity could indicate a greater likelihood of experiencing back pain (50). Additionally, economic development, lifestyle changes, physical activity decreases, and dietary changes affect LBP development (51).

Additionally, we revealed that the burden of LBP due to high BMI increases with age. The crude DALYs rates for all age groups experienced a significant increase from 1990 to 2021 across all SDI regions. The highest DALYs rates in high SDI regions were found in women aged 70–74 years and men aged 65–69 years, whereas the DALYs rates peaked among women aged 55–59 years and men aged 60–64 years in medium and low SDI regions. These outcomes indicate that the LBP burden related to high BMI is more substantial in the elderly than in younger demographics. This trend can be ascribed to several contributing factors. First, the mounting challenge of an aging global population caused a notable upsurge in the burden of LBP due to high BMI. Our decomposition analysis indicates that population growth and epidemiological shifts accounted for 63.08 and 42.51%, respectively, of the heightened burden of LBP due to high BMI on a worldwide scale. Second, older adults are more likely to become obese due to slower metabolic rates, reduced physical activity, chronic disease and medication effects, and psychological factors (52). Third, many countries have implemented effective weight reduction measures that are expected to alleviate the disease burden in younger populations to address this challenge. In particular, the Luton City Council in the United Kingdom (UK) has taken steps to restrict advertising for unhealthy foods and beverages, thereby reducing residents’ exposure, especially among children (53). The WHO has issued fresh guidelines advocating for adopting sweeping mandatory policies to safeguard children of all age groups from the promotional activities surrounding high-sugar, high-salt, and high-fat foodstuffs (54). Moreover, a multitude of nations and territories have enforced levies on sugar-sweetened beverages (SSBs) to curtail their consumption and secure funds for public health endeavors. Studies from Mexico and the United States have demonstrated a contraction in SSBs sales and a concurrent upsurge in the sales of non-taxed drinks, such as water (55, 56). The UK’s imposition of a sugar tax has likewise prompted a decrease in the sugar content of beverages (57). The initiative of the United States to eliminate artificial trans fats from the food supply represents a monumental policy measure (58).

The burden of LBP caused by high BMI significantly differs globally, and this variation may be caused by a multitude of factors. Data from 2021 exhibit that the ASDR of LBP due to high BMI varies by nearly 17 times between countries. The ASDR is especially severe, particularly in some countries in the South Pacific, as well as in most countries in North America and Northern Europe. This may be associated with the dietary habits of residents in these regions, who may prefer foods that are high in calories, fat, and sugar. Moreover, the genetic heritage of Americans as descendants of nomadic tribes may predispose them to store fat as a strategy for dealing with food shortages. The history of colonization may have resulted in shifts in dietary practices in the South Pacific, thereby affecting the weight and health of the local population (59). Compared to sub-Saharan Africa and South Asia, Latin America has a higher ASDR for obesity-related lower back pain. This phenomenon may be attributed to the region’s rapid increase in obesity rates, unhealthy lifestyle habits, socio-economic challenges, the widespread popularity of processed foods, and the uneven distribution of medical resources (60). The EAPC values from the GBD study indicate that the disease burden in nearly every country increased to some degree from 1990 to 2021, with the most notable rises in Cambodia and North Korea. This could be caused by better economic conditions, resulting in diets that include more high-calorie foods, as well as the traditional diet of the North Korean ethnic group, which is known for its unique and rich ethnic flavors (61). From the EAPC, it can be observed that the trend of low back pain burden in Latin America is relatively stable or has a slower growth rate, while regions such as sub-Saharan Africa exhibit higher EAPC values. This discrepancy may reflect significant progress in the implementation of public health policies or therapeutic measures to address obesity issues in Latin America (62). In contrast, regions with higher EAPC values may experience a continuous increase in the burden of obesity-induced low back pain due to factors such as rapid urbanization, population aging, and changes in lifestyle (63). Furthermore, the quality and comprehensiveness of health data in different regions may also influence these differences, as the accuracy and timeliness of data are crucial for accurately reflecting the actual situation. Our research determined a significant negative correlation between EAPC and ASDR. There is significant variation in the burden of low back pain across different regions; some affluent countries have a higher actual burden than anticipated, while some Asian countries have a lower burden than expected. This indicates that countries should focus on and implement targeted prevention and intervention strategies according to their unique characteristics.

We conducted a frontier analysis at the national level using ASDR and SDI data. Noteworthily, excellent management capabilities have been demonstrated in countries with lower SDI (< 0.5), such as Somalia, Lao People’s Democratic Republic, Timor-Leste, Papua New Guinea, and Yemen. These countries are typical examples that showcase effective strategies for improving health outcomes under resource-limited conditions despite limited resources in these countries. In particular, Somalia has implemented the Somalia Nutrition Strategy (2020–2025), to address all forms of malnutrition, including overweight and obesity (64). Research in Timor-Leste has revealed the prevalence of underweight, overweight, and obesity statuses, along with associated factors among the 15–49-year-old demographic, potentially providing data to inform policy development (65). Conversely, countries with a higher SDI (> 0.85), such as the Netherlands, Germany, Canada, the United States, and Iceland, demonstrate a relatively more pronounced effective disparity in terms of development level. The German Health Update Study (GEDA 2019/2020-EHIS) reveals a significant proportion of overweight and obese adults in Germany, particularly within the 45–64-year age bracket. The study emphasizes the substantial potential for obesity prevention, requiring a focus on preventive initiatives to alter individual health behaviors and implement measures to reduce social health inequalities. This objective has yet to be fully realized, whereas Germany’s sustainable development strategy is aimed at curbing the increasing trend of obesity among adults (66). The Netherlands has incorporated measures against obesity into its national prevention strategy, which includes providing healthier food options in at least 2,500 sports clubs and 950 schools, with a commitment for half of the hospitals to provide healthier food choices by 2025. Additionally, the Netherlands has initiated various community-based intervention and movement programs targeting obesity, such as “CooL (Coaching on Lifestyle)” and the “National Prevention Strategy 2018.” However, there is scope for further improvement in managing the burden of LBP attributable to increased BMI levels (67).

This study has several limitations to be considered when interpreting the results. First, a high BMI is defined as > 25 kg/m^2^, with 20–25 kg/m^2^ considered the lower detection limit. Future studies should establish more specific criteria for personalized analysis to improve the precision of the assessment and increase the credibility of the results. Second, the absence of accessible or high-quality data in certain regions may decrease data accuracy and delay information provision, thereby potentially causing overestimations or underestimations of the disease burden for specific regions or time frames. Third, the GBD study lacks prevalence data on LBP due to high BMI, constraining our capability to integrate this vital information into our analysis. Finally, the global prevalence and childhood obesity could not be analyzed due to the lack of data despite their increase and significance. These limitations indicate a need for more careful and comprehensive approaches to data collection and analysis in future studies.

## 5 Conclusion

In summary, this study uses the most recently published GBD 2021 database to provide a comprehensive update on the global trends and burden of LBP caused by high BMI up to the year 2021. An increasing trend in LBP caused by high BMI globally was observed for 30 years, from 1990 to 2021, with a projected continuation of this increase, emphasizing its status as a significant and heretofore underappreciated element of the global disease burden. Engaging in consistent monitoring and appraisal of BMI has become imperative to develop region- and country-sensitive strategies that accommodate local particularities. The results of this study bring into focus the urgency of taking measures to alleviate the health burden due to high BMI and to improve the quality of life for the global population.

## Data Availability

The datasets presented in this study can be found in online repositories. The names of the repository/repositories and accession number(s) can be found in this article/Supplementary material.
