# Supplementary material for: Low back pain trends attributable to high body mass index over the period 1990–2021 and projections up to 2036
Source: Front Nutr. 2025 Jan 21;11:1521567. doi: 10.3389/fnut.2024.1521567 (PMC11790459; doi:10.3389/fnut.2024.1521567)
Supplement: Supplementary file 1 [file Data_Sheet_1.zip › Data Sheet 1/Supplementary Figures.docx]

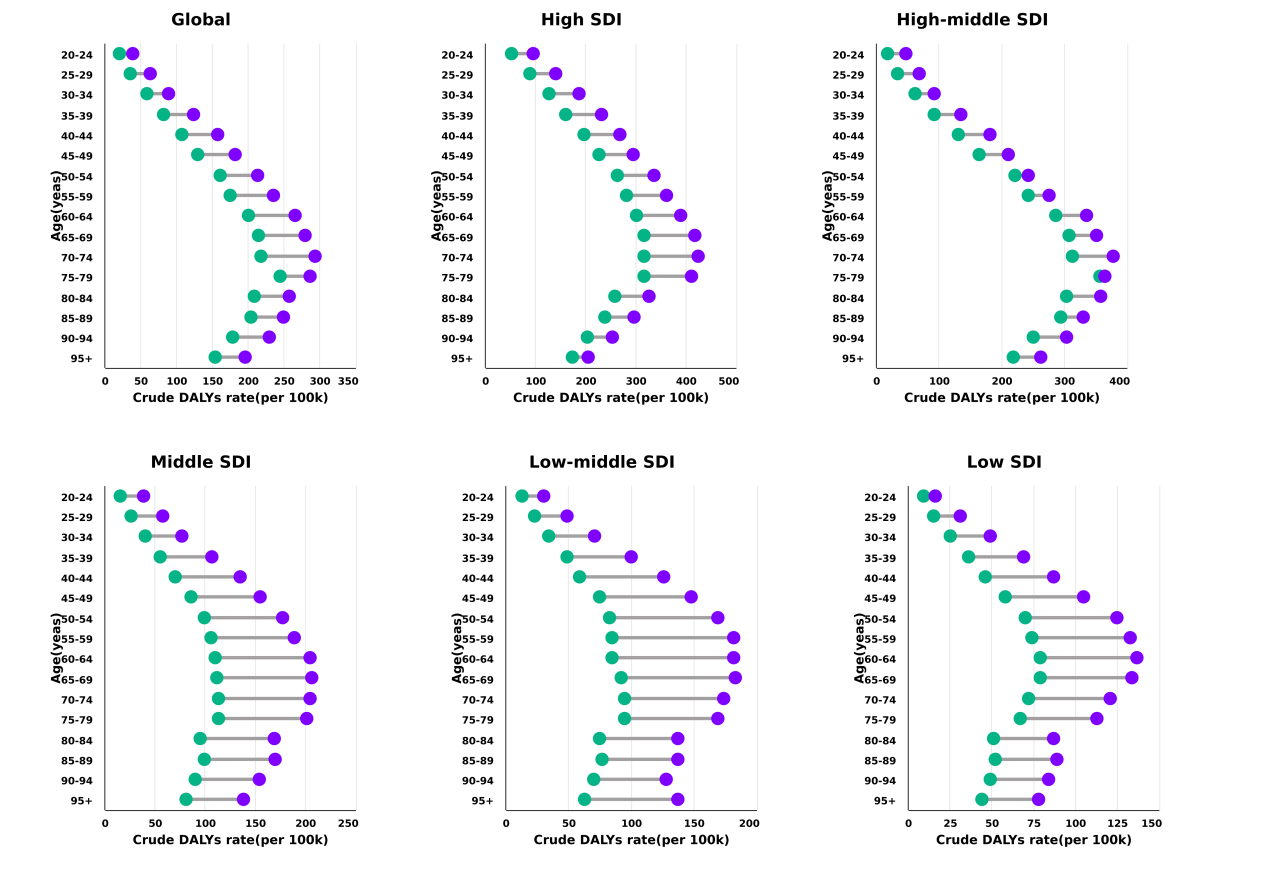


Supplementary Figure S1. The dumbbell chart shows the change in the crude DALYs rate of

various age groups for low back pain attributed to high body mass index in different regions from 1999 to 2021. DALYs, disability-adjusted life-years.Green for 1990, purple for 2021


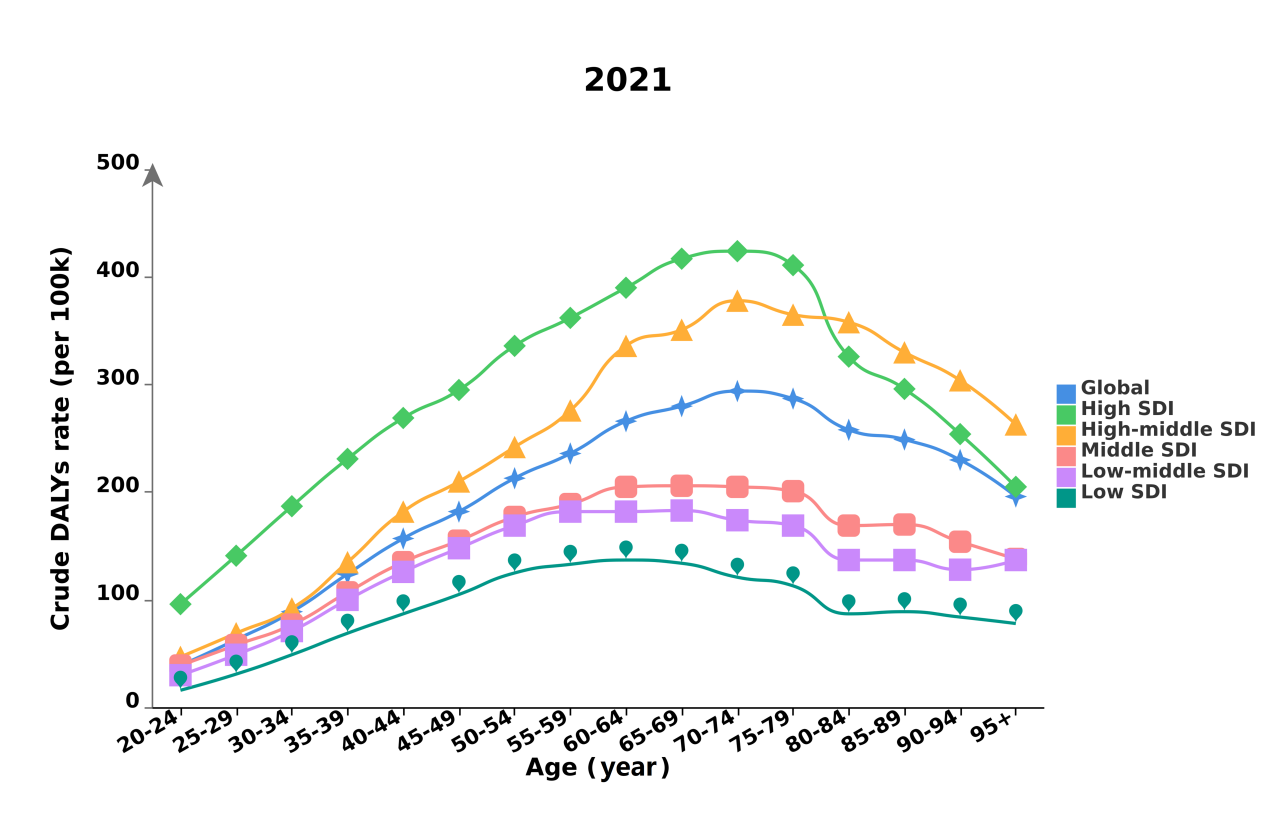


Supplementary Figure S2. Temporal trend of crude deaths rate and DALYs rate of low back pain attributed to low back pain from young to old people in different SDI regions. Different colored

curves represent the results of different regions. DALYs, disability-adjusted life-years; SDI, socio-demographic index.
